# Metabolic Coupling Determines the Activity: Comparison of 11β-Hydroxysteroid Dehydrogenase 1 and Its Coupling between Liver Parenchymal Cells and Testicular Leydig Cells

**DOI:** 10.1371/journal.pone.0141767

**Published:** 2015-11-03

**Authors:** Xingwang Li, Guoxin Hu, Xiaoheng Li, Yi-Yan Wang, Yuan-Yuan Hu, Hongyu Zhou, Syed A. Latif, David J. Morris, Yanhui Chu, Zhiqiang Zheng, Ren-Shan Ge

**Affiliations:** 1 The Second Affiliated Hospital & Yuying Children's Hospital, Wenzhou Medical University, Wenzhou, ZJ 325000, PR China; 2 Research Academy of Reproductive Biomedicine, Wenzhou Medical University, Wenzhou, ZJ 325000, PR China; 3 Department of Pathology and Laboratory Medicine, The Miriam Hospital, Brown University School of Medicine, Providence, RI 02906, United States of America; 4 Heilongjiang Key Laboratory of Anti-fibrosis Biotherapy, Mudanjiang Medical University, Mudanjiang, Heilongjiang, PR China; 5 Population Council, 1230 York Avenue, New York, NY 10065, United States of America; Casey Eye Institute, UNITED STATES

## Abstract

**Background:**

11β-hydroxysteroid dehydrogenase 1 (11β-HSD1) interconverts active 11β-hydroxyl glucocorticoids and inactive 11keto forms. However, its directionality is determined by availability of NADP+/NADPH. In liver cells, 11β-HSD1 behaves as a primary reductase, while in Leydig cells it acts as a primary oxidase. However, the exact mechanism is not clear. The direction of 11β-HSD1 has been proposed to be regulated by hexose-6-phosphate dehydrogenase (H6PDH), which catalyzes glucose-6-phosphate (G6P) to generate NADPH that drives 11β-HSD1 towards reduction.

**Methodology:**

To examine the coupling between 11β-HSD1 and H6PDH, we added G6P to rat and human liver and testis or Leydig cell microsomes, and 11β-HSD1 activity was measured by radiometry.

**Results and Conclusions:**

G6P stimulated 11β-HSD1 reductase activity in rat (3 fold) or human liver (1.5 fold), but not at all in testis. S3483, a G6P transporter inhibitor, reversed the G6P-mediated increases of 11β-HSD1 reductase activity. We compared the extent to which 11β-HSD1 in rat Leydig and liver cells might be coupled to H6PDH. In order to clarify the location of H6PDH within the testis, we used the Leydig cell toxicant ethane dimethanesulfonate (EDS) to selectively deplete Leydig cells. The depletion of Leydig cells eliminated *Hsd11b1* (encoding 11β-HSD1) expression but did not affect the expression of *H6pd* (encoding H6PDH) and *Slc37a4* (encoding G6P transporter). *H6pd* mRNA level and H6PDH activity were barely detectable in purified rat Leydig cells. In conclusion, the availability of H6PDH determines the different direction of 11β-HSD1 in liver and Leydig cells.

## Introduction

Glucocorticoids (GCs) have a wide range of physiological and pharmacological roles in mammalian functions [1, 2]. Excessive GCs under conditions such as stress and Cushing’s disease cause a spectrum of clinical features including metabolic syndrome and reduced fertility [3].

Intracellular levels of GCs (corticosterone, CORT, in rats, and cortisol in humans) are regulated by 11β-hydroxysteroid dehydrogenase (11β-HSD) that has two known isoforms, type I (11β-HSD1) and type II (11β-HSD2). 11β-HSD1 is an NADP^+^/NADPH dependent oxidoreductase, catalyzing the interconversion of 11β-hydroxyl steroids (CORT and cortisol) and 11-keto steroids (such as 11-dehydrocorticosterone, 11DHC, in rats, and cortisone in humans) and is most abundantly expressed in GC target tissues such as testis, liver, and fat [4]. In rat testis, 11β-HSD1 is only expressed in the Leydig cell, which produces testosterone [5, 6]. The expression level of 11β-HSD1 in the rat Leydig cell is the highest among all cell types, and its level was about 4 fold higher than that in liver cells [7]. 11β-HSD1 is a low-affinity high capacity enzyme with a K_m_ of 300–500 nM [4]. Its direction of catalysis depends on the cell type and intracellular milieu. For example, when a plasmid containing the entire coding region of 11β-HSD1 gene (*Hsd11b1*) was transiently transfected into two different cell lines, CHOP and COS1, oxidase activity was predominant in the former whereas reductase activity was higher in the latter [8].

The catalytic direction of 11β-HSD1 depends on the redox potential set by the NADP^+^/NADPH cofactor ratio [9]. Recently, it has been shown that the NADP^+^/NADPH ratio in smooth endoplasmic reticulum luminal space was modulated by hexose-6-phosphate dehydrogenase (H6PDH) activity [9, 10]. For example, in liver cell smooth endoplasmic reticulum luminal space, H6PDH catalyzes the synthesis of NADPH thereby raising the intra-luminal level of the cofactor and this may favor the reductase activity of 11β-HSD1 observed in several tissues [9, 10]. H6PDH uses glucose-6-phosphate (G6P) as a substrate and NADP+ as cofactor, to produce 6-phosphogluconate and NADPH. In transgenic mice with H6PDH null mutation, negligible NADPH is generated, and thus 11β-HSD1 in the mutated mouse liver functions primarily as an oxidase [11].

We previously demonstrated that unlike the enzyme in liver cell the 11β-HSD1 in rat Leydig cells was a primary oxidase [6]. Although the 11β-HSD1 direction was demonstrated to be determined by the H6PDH, its direction in rat and human Leydig cells is unclear. In the present study, we investigated the possible mechanism that determines 11β-HSD1 directionality in these two cell types.

## Materials and Methods

### Chemicals and animals

[1, 2, 6, 7-^3^H] Corticosterone (^3^H-CORT) and [1, 2, 6, 7-^3^H] cortisol (^3^H-cortisol) were purchased from Dupont-New England Nuclear (Boston, MA). ^3^H-11Dehydrocorticosterone (^3^H-11DHC) and ^3^H-cortisone were prepared from labeled ^3^H-CORT or ^3^H-cortisol as described earlier and the purity was over 98%, which was determined by the radiometry [12]. Cold CORT, 11DHC, cortisol and cortisone were purchased from Steraloids (Newport, RI). S3483 was a gift from Aventis Pharma Deutschland. Male Sprague-Dawley rats (250–300 g) were purchased from Charles River Laboratories (Wilmington, MA). Human liver microsomes were purchased from Gentest (Woburn, MA). Human male testes were obtained from National Disease Research Interchange (Philadelphia, PA). The ethane dimethanesulfonate (EDS) was a gift from Dr. L. Earl Gray, U.S. Environmental Protection Agency, Research Triangle Park, NC; purchased from Radion Co.).

### Animal treatments

Male Sprague-Dawley rats (90-day-old) were divided into 6 groups of 6 animals. 30 rats were intraperitoneally given 75 mg/Kg EDS, a chemical that specifically eliminates Leydig cells in adult rat testis [13]. The other 6 animals received the same volume of vehicle (1:4, v/v, DMSO/water) and the animals were killed 1 h later as control. Rats were killed 4, 7, 14, 35 and 90 days post EDS. Rats were euthanized by asphyxiation with CO_2_. One testis each rat per group was punched three holes using a needle and fixed in Bouin solution overnight at 4°C, and stored in 70% ethanol at 4°C until further processing for immunohistochemistry. The contralateral testis from each animal other animals was frozen in liquid nitrogen for RNA analysis. The animal protocol was approved by the Institutional Animal Care and Use Committee of the Rockefeller University.

### Cell isolation

Testes were removed for purification of Leydig cells. Leydig cells were purified from rats as described previously [14]. Purities of Leydig cell fractions were evaluated by histochemical staining for 3β-hydroxysteroid dehydrogenase (3β-HSD), with 0.4 mM etiocholanolone as the steroid substrate [15]. More than 95% adult Leydig cells were intensely stained. Isolation of liver parenchymal cells was performed according to previously published method [6]. Livers of male rats were perfused *in situ* with a calcium-free buffer, then dispersed by a solution containing 0.05% collagenase, and parenchymal cells were purified by density gradient centrifugation in Percoll. The purity of parenchymal cells in the final suspension was assessed by judging the uniformity of cell size in hemocytometer counts and was typically over 95%. Four isolations of Leydig cells or liver cells were performed.

### Preparation of microsomal protein

Microsomal preparations of rat Leydig and liver cells as well as human testes were prepared as described previously [6]. Pellets were resuspended. The protein contents of microsomes were measured using the Bio-Rad protein assay solution with bovine serum albumin as a standard according to the manufacturer's instructions. The intactness of the microsomal vesicles was checked by measuring the latency of UDP-glucuronosyl transferase activity [16]. Latency was > 95% in all microsomal preparations. Microsomes were used for measurement of 11β-HSD1 and H6PDH activities. The orientation of the microsomal vesicles was analyzed by measuring the 11β-HSD1 reductase activity during the course of time with or without adding the pore-forming agent alamethicin (0.1 mg/mg protein) to allow the free access of the cofactor to the intraluminal enzyme as described [17].

### Primer selection and real-time PCR (Q-PCR)

All primers in this study were chosen using a sequence analysis software package (Primer 3, Whitehead Institute for Biomedical Research, Cambridge, MA) following guidelines for internal stability. Forward and reverse primers were in different exons to minimize the effects of possible DNA contamination. The primers for genes of 3β-HSD1 (*Hsd3b1*) and 11β-HSD1 (*Hsd11b1*) and 11β-HSD2 (*Hsd11b2*) were reported in the previous study [18]. For the internal standard, primers to ribosomal protein S16 (*Rps16*) were as described previously [18]. The primers for H6PDH (*H6pd*) and G6P transporter (*Slc37a4*) were (forward and reverse pairs): 5'-ACCGGGGACCTAGCTAAAAA-3', 5’-CTCAAGATTATCCCAGAGG-3’ for *H6pd* and 5’-TTGGACTCCGAAATCTGGAC-3’, 5’-GGTCACTGTTACCCGGAAGA-3’ for *Slc37a4*. First strand cDNAs, synthesized using total RNA from adult rat testis as described previously [18], were used as templates for PCR. Real-time PCR (qPCR) was carried out in a 25-μl volume using a 96-well plate format using the SYBR Green PCR Core Reagents purchased from Applied Biosystems (Foster City, CA) by a standard curve method as described previously [18]. Primer titration was performed and the concentration of 300 nM was selected. Fluorescence was detected on an ABI 7700 system (PE Applied Biosystems). Each sample was run in duplicate. The levels were normalized to *Rps16*.

### Immunohistochemistry

Testis sections were processed for immunohistochemistry (Vectastain, Elite, ABC Kit, PK-6101, Vector Laboratories, Inc., Burlingame, CA) according to the manufacturer’s instructions. The primary antibody to 11β-HSD1 was a rabbit polyclonal antibody [19]. Endogenous peroxidase was blocked with 0.5% H_2_O_2_ in methanol for 30 min. The sections were then incubated with anti-11β-HSD1 (diluted 1:1000) for 1 h at room temperature. The antibody-antigen complexes were visualized with diaminobenzidine (Peroxidase Substrate Kit, SK-4100, Vector Laboratories, Inc.). The sections were counterstained with Mayer’s hematoxylin, dehydrated in graded concentrations of alcohol, and cover-slipped with resin (Permount, SP15–100, Fisher Scientific Co.). Control sections were incubated with non-immune rabbit IgG using the same working dilution as the primary antibody.

### 11β-HSD assay

11β-HSD activity assay tubes contained 25 nM CORT. This concentration was chosen based on its level within the range of physiological level. [^3^H]-CORT or [^3^H]-11DHC were used as substrates to measure rat 11β-HSD1 oxidase or reductase activity, respectively. [^3^H]-Cortisol or [^3^H]-cortisone were used as substrates to measure human 11β-HSD1 oxidase or reductase activity, respectively. Intact rat Leydig or liver cells were incubated with S3483 (0.01–100 μM in the final concentration). S3483 was dissolved in ethanol and was added to cells, the final concentrations of ethanol of 0.1%, at which concentration ethanol did not affect cell viability and 11β-HSD1 activity) for 30 min and then [^3^H]-CORT or [^3^H]-11DHC were added into the cells and incubated for 0.5–2 hrs for measuring either 11β-HSD1 oxidase or reductase activity, using endogenous cofactor. For microsomal preparation, the Leydig or liver cell or human liver as well as testis microsomes were incubated with substrates, NADPH or G6P. Generally, 0.2 mM NADPH was used because the average level of NADPH in cells is around 0.1–0.16 mM [20] and 0.2 mM NADPH is saturated level to guarantee 11β-HSD1 to maximally metabolize the steroid substrate [20]. In some experiments, S3483, the specific inhibitor of G6P transporter, was used to inhibit this transporter activity 10 min before incubation. Then the reaction mixture was incubated at 37°C for 10–30 min. The reactions were stopped by adding 2 ml ice-cold ether. The steroids were extracted, and the organic layer was dried under nitrogen. The steroids were separated chromatographically on thin layer plates in chloroform and methanol (90:10, v/v), and the radioactivity was measured using a scanning radiometer (System AR2000, Bioscan Inc., Washington, DC). The percentage conversion of 11β-hydroxyl steroid to 11-keto steroid was calculated by dividing the radioactive counts identified as 11-keto steroid by the total counts to calculate 11β-HSD1 oxidase, and vice versa for the reductase.

### H6PDH assay

The H6PDH assay was described previously [21]. In brief, H6PDH activity was determined spectrophotometrically by monitoring NADPH generated at 25°C. The assay mixture contained the components in a total volume of 1 ml; 80 mM glycine-NaOH buffer (pH 10.5, 1% Triton X-100)/ 200 μM G6P/80 μM NADP+ in 1% triton X. The reaction was started by the addition of 10 μl enzyme solution.

### NADP+ and NADPH assay

The NADP+ and NADPH assay was described previously [22]. The method used the influence of absorbance at 340 nm by NADPH not NADP+. In brief, the homogenates of purified liver cells and Leydig cells were separated respectively into three parts. Part one, the untreated one, was measured the absorbance at 340 nm. Part two, after treated with enzyme that converted NADP+ to NADPH, was measured the absorbance at 340 nm. Part three, after treated with an enzyme that converted NADPH to NADP+, was measured absorbance at 340 nm. Then the NADP+ and NADPH contents were calculated.

### Cell viability assay

Trypan blue was used to test the viability of cells as described previously [23]. Leydig cells or liver cells were treated with different concentrations of S3483 or ethanol. Cell suspensions (20 μl) were mixed with equal volumes of 0.4% trypan blue for 5 min at room temperature. Ten μl of cell mixture was placed on a hemocytometer to count the number of viable (unstained) and dead (stained) cells. The average number of unstained cells in each quadrant was calculated. The percentage of living cells was defined as the number of viable cells divided by the total number (dead plus viable) of cells.

### Western blot

Western blot analysis was performed as previously described [24]. In brief, 40 μg protein from each sample was used. Samples were denatured, electrophoresed and transferred into a membrane. The membrane was incubated with 11β-HSD1 polyclonal rabbit antibody (kindly raised in house as described [24], dilution 1:1000). The membrane was then washed and incubated with a 1:1000 dilution of second antibody that was conjugated to horseradish peroxidase (anti-rabbit IgG, HRP-linked whole antibody produced in goat and anti-goat IgG, HRP-linked whole antibody produced in rabbit (Abcam Inc., Cambridge, MA). The washing step was repeated, and immunoreactive bands were visualized by chemiluminescence using a kit (ECL, GE Healthcare, UK). Western blot showed that the 11β-HSD1 in microsomes was intact (S1 Fig).

### Statistics

Each experiment was repeated three to four times. Data were subjected to analysis by a Student t-test when two groups were compared. One-way ANOVA analysis followed by ad hoc Turkey’s multiple comparisons to identify significant differences between two groups, when three and more groups were analyzed. All data are expressed as means SEM. Differences were regarded as significant at *P* < 0.05.

## Results

### 11β-HSD1 in intact liver and Leydig cells

**Fig 1:**
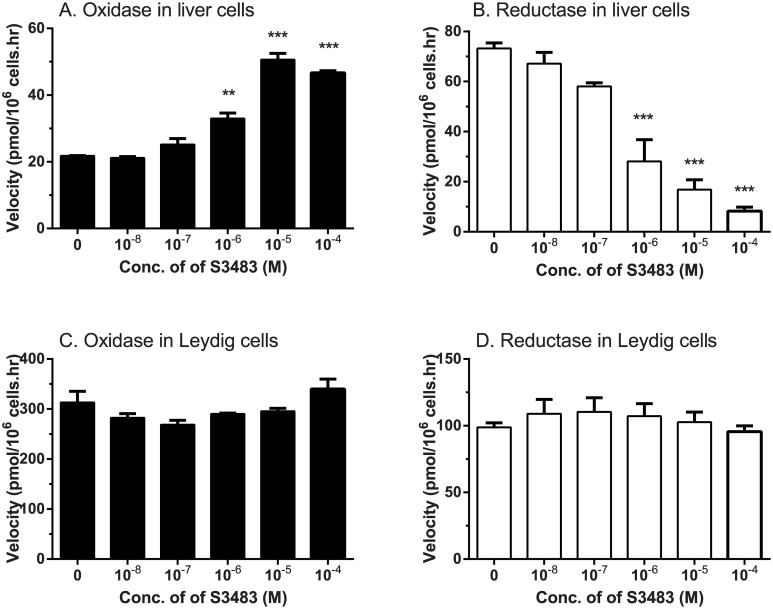
Effects of G6P transporter inhibitor S3483 on intact rat liver and Leydig cell 11β-HSD1 direction. 0.015 × 10^6^ liver or 0.025 × 10^6^ Leydig cells were used to measure 11β-HSD1 oxidase (CORT→11DHC) or 0.015 × 10^6^ liver or 0.05 × 10^6^ Leydig cells for reductase (11DHC→CORT) activities when the substrates were incubated with the cells for 30–120 min. S3483 was added into cells with concentration of 0.01–100 μM 10 min before addition of substrates. Mean ± SEM, n = 3~7. The asterisks designate significant differences compared to control (CON, no S3483) at *P < 0.05, **P < 0.01, and *** P < 0.001.

The directionality of 11β-HSD1 in intact rat liver parenchymal cells and Leydig cells was determined using endogenous cofactor (Fig 1). In our experiments, we first performed the time-course reaction in order to determine the linear range of 11β-HSD1 oxidase and reductase. We found that the reductase activity was in linear reaction during 0–1 h and the oxidase activity was in linear reaction during 0–4 h. In liver cells, 11β-HSD1 reductase activity was 3 fold higher than oxidase activity (Fig 1A). The percentage of 11β-HSD1 reductase activity in 0.015 × 10^6^ liver cells after 0.5 h incubation was 20.1%, while that of 11β-HSD1 oxidase activity in 0.015 × 10^6^ liver cells after 2 h incubation was 14.6%. However, in intact Leydig cells, the percentage of 11β-HSD1 oxidase activity in 0.025 × 10^6^ Leydig cells after 30-min incubation was 16.29%, with a velocity of 313.0 pmol/10^6^ cells.hr, while that of 11β-HSD1 reductase activity in 0.05 × 10^6^ cells after 2 h incubation was 41.79% with a velocity of 98.76 pmol/10^6^ cells.hr. This showed that 11β-HSD1 oxidase was significantly higher than reductase activity (Fig 1C and 1D). To further test whether H6PDH rendered this difference, G6P transporter inhibitor S3483 was used. Our rationale was that if the reductase activity of 11β-HSD1 was metabolically coupled to H6PDH, then availability of the substrate for H6PDH, G6P, would also be important. Addition of S3483 significantly increased the oxidation and decreased the reduction of 11β-HSD1 in intact liver cells (Fig 1A and 1B). This is what would be expected because the metabolic coupling between H6PDH and 11β-HSD1 has previously been shown [9, 10]. In contrast, addition of S3483 (up to 100 μM) to intact Leydig cells had no effect on either oxidation or reduction by 11β-HSD1 (Fig 1C and 1D). When 100 μM S3483 was used, it had a little bit cytotoxicity (data not shown), while other concentrations of S3483 did not affect both liver and Leydig cell viability (data not shown).

### 11β-HSD1 in liver and Leydig cell microsomes

**Fig 2:**
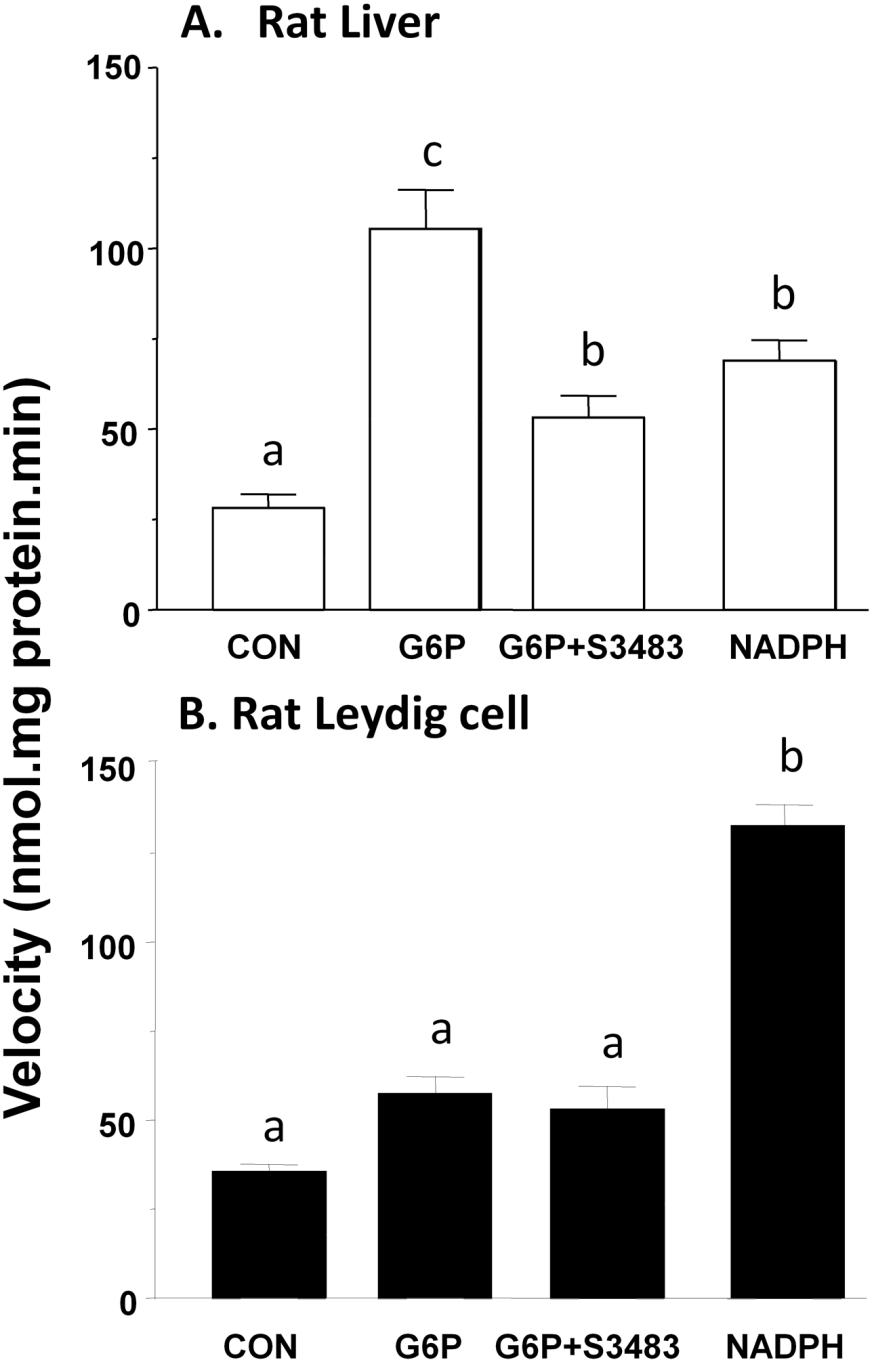
Effects of G6P and NADPH on rat liver and Leydig cell microsomal 11β-HSD1 reductase activities. 11β-HSD1 reductase activity was measured using the microsomes of rat liver cells (Panel A) or Leydig cells (Panel B). Mean ± SEM, n = 5. Identical letters designate that there were no significant differences between two groups at P < 0.05.

Rat liver microsome 11β-HSD1 reductase activity was measured in the presence of 0 to 400 μM G6P. G6P concentration-dependently increased liver microsome 11β-HSD1 reductase activity with 100 μM as a saturated concentration threshold (S2 Fig). However, addition of 0 to 400 μM G6P to rat Leydig cell microsomes had almost no effect on the velocity of the reaction (S2 Fig). The reductase activity of 11β-HSD1 in rat liver microsomes was stimulated significantly by 200 μM G6P after 10 min incubation. The addition of 1 μM S3483 (Fig 2A) reversed the stimulation by G6P. S3483 alone did not affect rat liver 11β-HSD1 oxidase and reductase activity when it was used up to 100 μM (S3 Fig). In contrast, G6P had no effects of 11β-HSD1 in rat Leydig cell microsomes (Fig 2B). We measured the NADP+ and NADPH levels in intact liver and Leydig cells, and found that both cells had the similar levels of NADP+ and NADPH (S1 Table). We further added 0.2 mM NADPH to rat liver and Leydig cell microsomes. NADPH increased 11β-HSD1 reductase activity by 2 folds, while NADPH stimulated 11β-HSD1 reductase activity in rat Leydig cell microsomes by 4 folds (Fig 2). We infer that rat Leydig cell 11β-HSD1 reductase is more dependent on NADPH than G6P.

Rat liver microsome (2 μg) or Leydig cell microsome (0.3 μg) was incubated with 25 nM 11-dehydrocorticosterone for 30 min. The percentage of conversion of corticosterone into 11-dehydrocorticosterone was calculated. G6P dose-dependently increased the activity of 11β-HSD1 reductase in the liver microsome.

### 11β-HSD1 in human liver and testis microsomes

**Fig 3:**
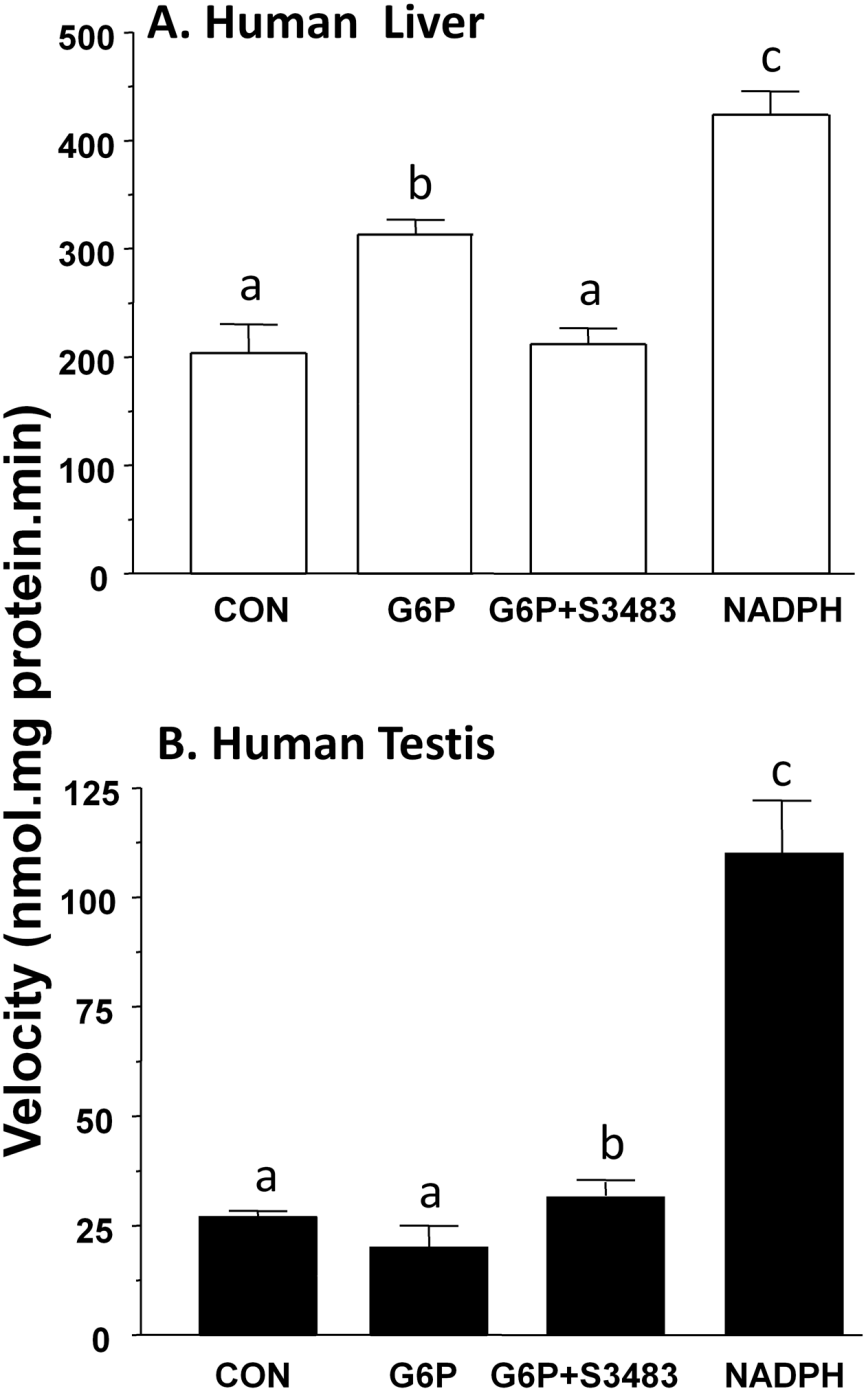
Effects of G6P and NADPH on human liver and testis microsomal 11β-HSD1 reductase activities. 11β-HSD1 reductase activity was measured using the microsomes of human liver (Panel A) or testis (Panel B). Mean ± SEM, n = 4. Identical letters designate that there were no significant differences between two groups at P < 0.05.

11β-HSD1 has been identified in human liver and testis [25, 26]. We investigated whether 11β-HSD1 reductase activities in human liver and testis microsomes depended on G6P or NADPH. In our hands, 200 μM G6P increased 11β-HSD1 reductase activity in human liver microsomes by 50% (Fig 3A), and 1 μM S3483 reversed the stimulation of G6P. 11β-HSD1 reductase activity in human testis microsomes, as in rat Leydig cell microsomes, was not stimulated by G6P (Fig 3B) and S3483 had no effects. 0.2 mM NADPH increased 11β-HSD1 reductase activity in human liver microsomes by 2 folds, while NADPH increased 11β-HSD1 reductase activity in human testis microsomes by 4 folds. We infer that H6PDH activity is not metabolically coupled to 11β-HSD1 activity in human Leydig cells either.

### H6PDH levels in microsomes of rat liver and Leydig cell

H6PDH activity was assayed in microsomes prepared from rat liver, testis, liver parenchymal cells and Leydig cells, in order to assess the relative abundance among these sources. H6PDH activity was 18 nmol/mg.h in the liver microsome and 13 nmol/mg.h in the testis microsome (Table 1). When microsomes were prepared from purified cells after cell fractionation, H6PDH was enriched 2.5-fold, to 45 nmol/mg.h, in liver parenchymal cells. However, H6PDH activity was barely detectable in rat Leydig cells when compared to rat testis microsomes (Table 1). This indicates that H6PDH activity in rat Leydig cells is very low. We also measured NADP+ and NADPH levels in intact liver and Leydig cells and found that there was no difference between two cell types (S1 Table).

**Table 1:** H6PDH activity in rat liver, testis and liver parenchymal cell and Leydig cell microsomes.

Source of Microsomes	H6PDH activity (nmol/mg.hr)	P
**Liver**	17.67 ± 0.76 (n = 6)	a
**Testis**	12.60 ± 0.77 (n = 5)	b
**Liver cell**	44.80 ± 1.78 (n = 5)	c
**Leydig cell**	4.80 ± 0.49 (n = 5)	d

Similar letters showed no significant differences between groups.

### Hsd11b1, Hsd11b2, H6pd, and Slc37a4 expression levels in rat Leydig cells

**Fig 4:**
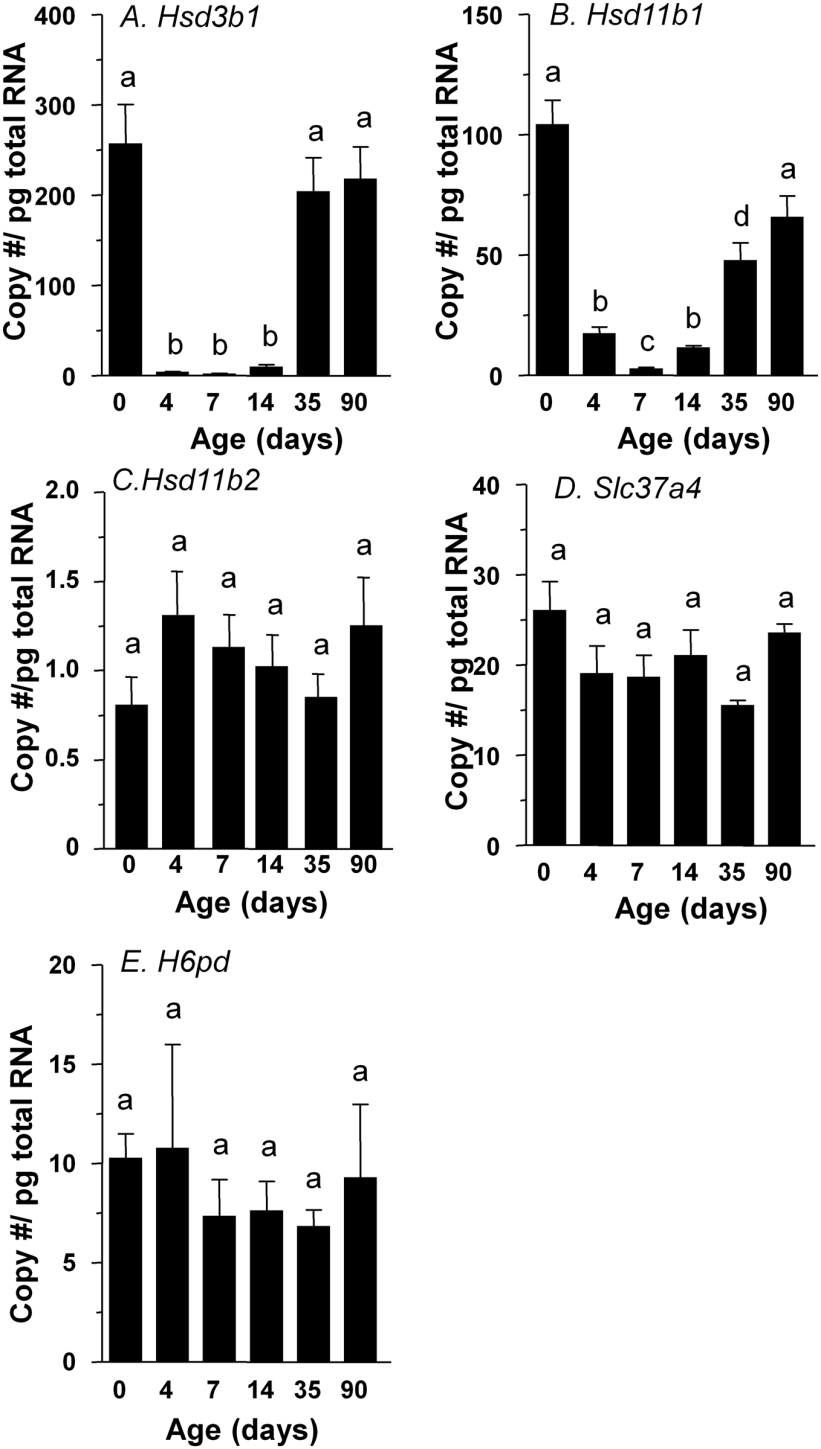
Real-time PCR analysis of *Hsd3b1*, *Hsd11b1*, *Hsd11b2*, *H6pd*, and *Slc37a4* in the testis at different days after EDS treatment. Mean ± SEM, n = 4~6. Identical letters designate that there were no significant differences between two groups at P < 0.05.

In order to detect the relative levels of *Hsd11b1* (encoding 11β-HSD1), *Hsd11b2* (encoding 11β-HSD2), *H6pd* (encoding H6PDH) and *Slc37a4* (encoding G6P transporter) in rat Leydig cells, an EDS-treated rat model, in which EDS specifically eliminates only Leydig cells [13], was used. Seven days post EDS, all Leydig cells were eliminated, as shown that there was undetectable *Hsd3b1* expression (Fig 4A). By 35 days post EDS, Leydig cell regeneration is in progress as shown by the *Hsd3b1* expression (Fig 4A). In this model, the presence of Leydig cell specific enzymes coincides closely with the absence or presence of Leydig cells.

**Fig 5:**
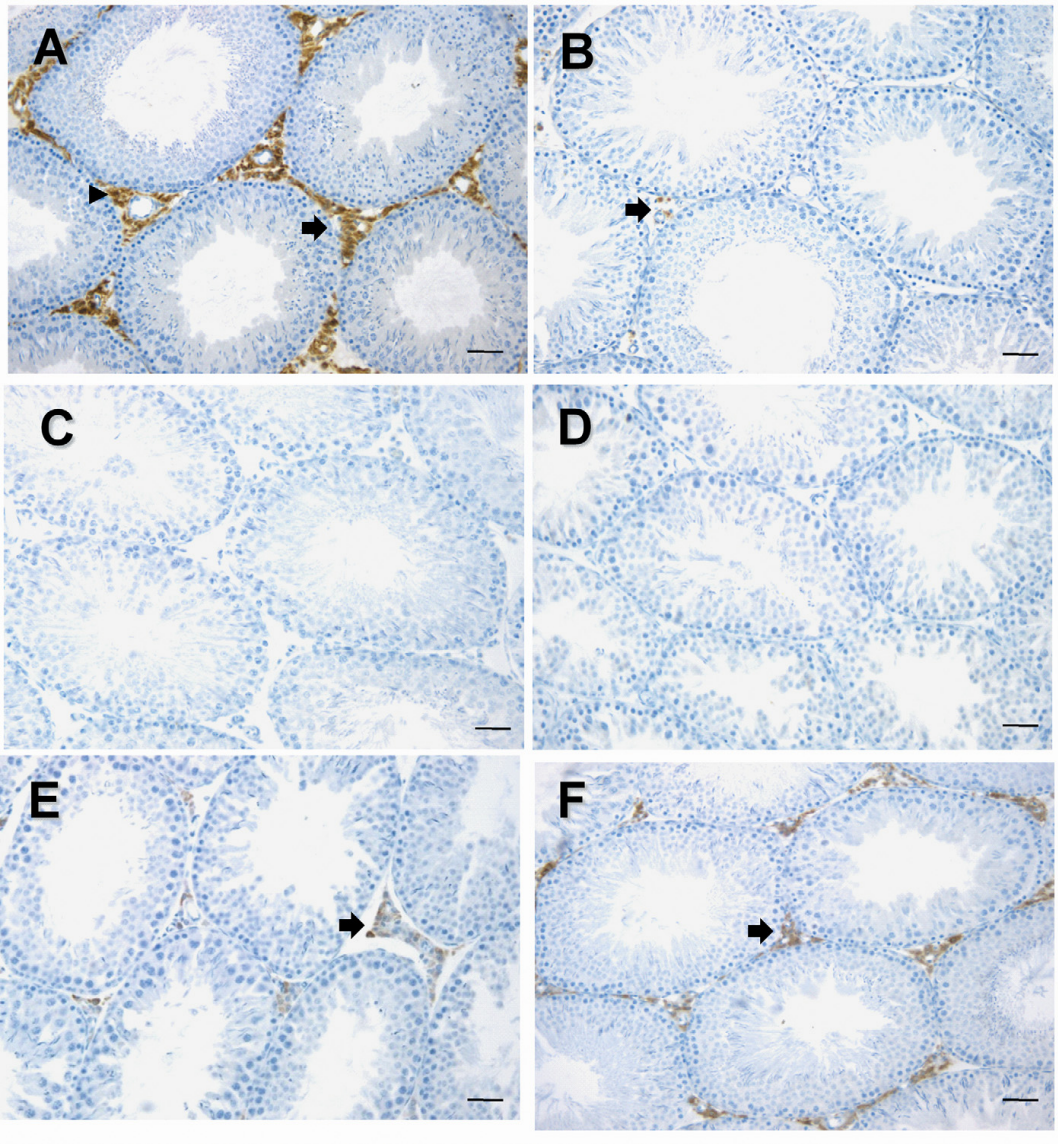
11β-HSD1 immunohistochemical staining in Leydig cells after EDS treatment. 90-day-old rats were injected with 70 mg/kg EDS. Panel A: 0; B: 4; C: 7; D: 14; E: 35; F: 90 days after EDS. Black arrow points to 11β-HSD1 positive cells. Bar = 50 μm.

**Fig 6:**
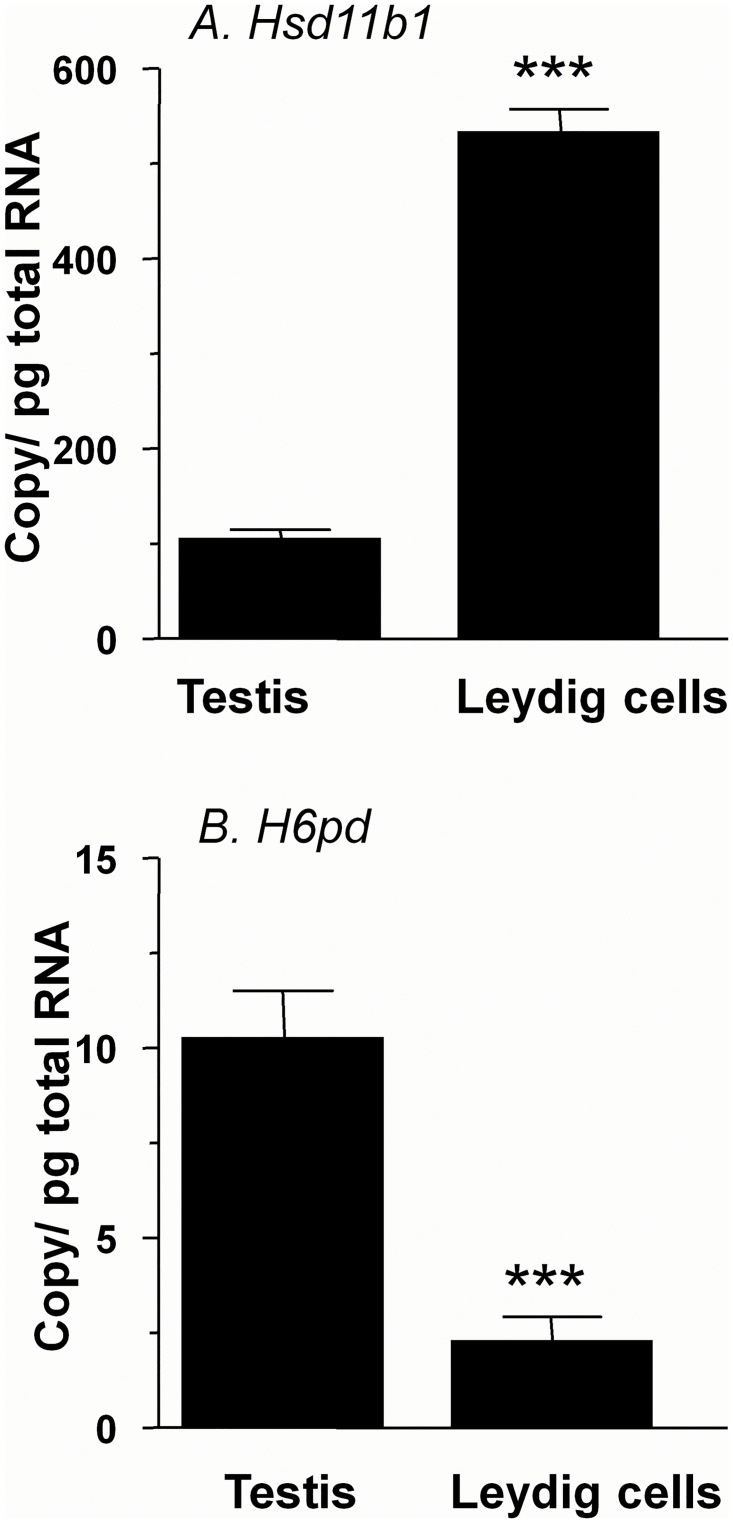
Comparison of *Hsd11b1* and *H6pd* in rat Leydig cells and testis. Panel A, *Hsd11b1* and Panel B, *H6pd*. Mean ± SEM, n = 4~6. *** designates significant differences between two groups at P < 0.001.

*Hsd11b1* signal was lower 4 days after the EDS injection, and was undetectable by 7 days after the injection of EDS (Fig 4B). 11β-HSD1 became detectable by 35 days after the EDS treatment (Fig 5E), and completely recovered by 90 days post EDS (Fig 4B). Using testis mRNA extracted from the same treatment groups, we analyzed relative amounts of mRNAs for *Hsd11b1*. We found that the amounts of *Hsd11b1* mRNAs fell when Leydig cells were depleted, and rose during the time of Leydig cell regeneration (Fig 4B). We did not see any changes of *Hsd11b2* expression levels after EDS treatment (Fig 4C). We then measured mRNAs that should be present for metabolic coupling between 11β-HSD1 and H6PDH, which are *H6pd* (encoding H6PDH) and *Slc37a4* (encoding G6PT). On the other hand, amounts of *H6pd* and *Slc37a4* mRNAs did not change even from 7 to 14 days post EDS when Leydig cells were completely absent in the testis (Fig 4D and 4E). This suggests that that *H6pd* and *Slc37a4* mRNAs are other testicular non-Leydig cells since the elimination of rat Leydig cells did not affect their levels. We further compared the steady state mRNA level of *Hsd11b1* and *H6pd* in purified rat Leydig cells from 90-day-old rat testis (Fig 6). *Hsd11b1* mRNA level in rat Leydig cells was 533.0 ± 24.5 vs. 104.0 ± 10.4 copies/pg RNA in age-matched rat testis, indicating that *Hsd11b1* is enriched in Leydig cells. *H6pd* mRNA level in rat Leydig cells was 2.26 ±0.63 vs. 10.2 ± 1.2 copies/pg RNA in age-matched rat testis, indicating that *H6pd* is enriched in non-Leydig cell fractions. The expression level of *H6pd* in rat Leydig cells is far lower than that of *Hsd11b1*, confirming that H6PDH cannot form an effective metabolic coupling in rat Leydig cells to render 11β-HSD1 as a primary reductase.

In the adult rat testis, 11β-HSD1 protein was intensely stained in Leydig cells by immunohistochemistry (Fig 5A). The staining intensity was lower 4 days after the EDS injection, and was undetectable by 7 to 21 days after the injection (Fig 5B–5D). 11β-HSD1 staining became detectable by 35 days after the EDS treatment (Fig 5E), and completely recovered by 90 days post EDS (Fig 5F).

## Discussion

The present study provided evidence that, in contrast to liver, the Leydig cell 11β-HSD1 was not coupled to H6PDH. G6P did not affect 11β-HSD1 reductase activity in human testis microsomes but significantly stimulated enzyme activity in human liver microsomes. Rat Leydig cell 11β-HSD1 was not affected by G6P. Leydig cell microsomal 11β-HSD1 reductase was more dependent on NADPH.

GCs are implicated in many physiological roles and pathogenesis of many diseases and are regulated at the pre-receptor level by the enzymes 11β-HSD1 and H6PDH [9, 10]. It has been reported that H6PDH is the key regulator of the directionality of 11β-HSD1 activity in liver and fat [9, 10]. H6PDH is a component of a smooth endoplasmic reticulum—in many cells, it converts G6P to 6-phosphogluconate, and generates NADPH [9, 10]. In transgenic mice lacking H6PDH, 11β-HSD1 in the liver and fat behaves dehydrogenase rather than a reductase. This provides strong evidence that 11β-HSD1 reductase activity depends on the participation of H6PDH [11].

In the present study, rat liver and particularly rat liver parenchymal cells had higher levels of H6PDH activity than Leydig cells (Table 1). This data corroborates with the mRNA levels of *H6pd* in rat liver compared to rat testis [27]. We observed a barely detectable amount of H6PDH activity in isolated rat Leydig cells. Furthermore, we did not find significant stimulation of 11β-HSD1 reductase activity in rat Leydig cell microsomes after addition of G6P (Fig 2). This difference of H6PDH activity could explain the directionality of 11β-HSD1 as a primary reductase in the liver cells while as a predominant oxidase in Leydig cells, because both cell types had similar levels of intracellular NADP+ and NADPH (S1 Table).

In intact cells, the substrate G6P stimulated liver 11β-HSD1 reductase activity robustly. It has been hypothesized that H6PDH is the key regulator of 11β-HSD1 reductase activity in many cell types [28]. However, H6PDH and 11β-HSD1 do not co-exist uniformly [27]. For example, in the kidney, H6PDH immunoreactivity was present in most cells except those of the papillary interstitium and a few convoluted and straight descending proximal tubules of superficial nephrons, while 11β-HSD1 immunoreactivity was present in almost all proximal tubules of the superficial cortical nephrons and most of the distal convoluted tubules [27]. This could explain why 11β-HSD1 in proximal tubules behaves a primary dehydrogenase [29]. In the testis, H6PDH was detected in interstitial cells and maturing and mature spermatozoa but no other germ cell stages or in Sertoli cells [23]. However, 11β-HSD1 was very abundant in interstitial cells [27], which we also show in the present study. Although it was reported that rat interstitial cells were stained by anti-H6PDH antibody [27], in the present study the H6PDH and G6P transporter mRNA levels were unchanged after elimination of Leydig cells by the EDS treatment. The reduction of *Slc37a4* is of uncertain significance, and could still reflect dilution of a signal in Leydig cells by alternative sources of signal in the testis. However, the lower activity of H6PDH reflects its negligible expression in rat Leydig cells. Therefore, we conclude that H6PDH level in Leydig cells is very low. We detected an amount of H6PDH activity in rat Leydig cells (Table 1). However, this low H6PDH activity may not form the coupling with 11β-HSD1. Low H6PDH activity may explain the predominance of 11β-HSD1 oxidase activity in rat Leydig cells as shown in our previous study [6] and in the present study.

Rat Leydig cells also contain 11β-HSD2. However, its expression level is only about 1/1000 of 11β-HSD1 [18]. 11β-HSD2 is a pure NAD+-dependent oxidase that metabolizes 11β-hydroxyl steroids into 11keto steroids. Although there is small amount of 11β-HSD2 in rat Leydig cells or human testis, this co-existence with 11β-HSD1 did not mask our study for the coupling between H6PDH and 11β-HSD1. We measured 11β-HSD1 reductase activity, which uses NADPH generated by H6PDH from luminal NADP+ in the microsomes (Figs 2 and 3). The reduction of luminal NADPH levels using H6PDH inhibitor S3483 reversed G6P-stimulated 11β-HSD1 reductase activity in both rat and human liver microsomes, indicating that 11β-HSD1 is coupled with H6PDH in both rat and human livers. This confirms the data from other research groups [17, 30, 31]. NADPH is a direct coenzyme of 11β-HSD1, however, NADPH in promoting the reductase activity of 11β-HSD1 was less effective than G6P in rat liver (Fig 2A), confirming that NADPH unlike G6P was difficulty to penetrate microsomal membrane. In liver cell microsomal membrane, there is a transporter for G6P to get into microsomal lumen, since the G6P transporter inhibitor S3483 partially abolished the effects of G6P in rat liver as shown in a previous study [17] and in the present study (Fig 2A). Interestingly, NADPH was more effective than G6P in human liver as shown in Fig 3A to stimulate 11β-HSD1 reductase activity. We speculated that this species difference could be contributed by the difference of amount of G6P transporter. Indeed, G6P could increase human liver 11β-HSD1 reductase activity by 50%, at the same concentration, S3483 completely inhibited this increase. However, S3483 did not affect rat Leydig cell, rat and human testis microsomal 11β-HSD1 reductase activity, indicating that H6PDH is not coupled with 11β-HSD1 in rat and human testes.

We demonstrated that unlike liver 11β-HSD1 reductase in Leydig cells was dependent on NADPH as shown by the significant increase of its activity in the presence of NADPH (Fig 2). However, it is still unclear that how NADPH can get into smooth endoplasmic reticulum in Leydig cells to activate the 11β-HSD1 reductase activity. Apparently, it is unlikely the intactness of microsomes since the intactness test showed >95% intactness of the Leydig cell and human testis microsome in the present study. However, it is true that microsomes were not absolute impermeabile to pyridine nucleotides. A study showed that in the presence of 0.2 mM NADP+ a time-dependent uptake was observed after 15 min incubation with microsomes and intravesicular NADP+ concentration reached one-third of the extravesicular one after 1 h incubation [32]. The 11β-HSD1 assay in microsomes in the present study was performed in 30 min, during which pyridine nucleotide might be enough to generate 11β-HSD1 activity. The directionality of 11β-HSD1 in human liver and Leydig cells have not been determined *in vivo*. However, our *in vitro* data using G6P transport inhibitor S3483 showed that S3483 could reverse the G6P-stimulated increase of 11β-HSD1 reductase activity in human liver microsomes but had no effects on the enzyme activity in human testis microsomes. This indicates that human 11β-HSD1 is rendered to be a primary reductase by H6PDH and that 11β-HSD1 and H6PDH is not metabolically coupled like that in the liver. However, there are abundant NADPH-generating steroidogenic enzymes in the smooth endoplasmic reticulum in Leydig cells, and it is possible that they are coupled together to render 11β-HSD1 direction. In this regard, we recently reported that 11β-HSD1 was coupled with 17β-HSD3 in rat Leydig cells, thus the formation of NADP+ by 17β-HSD3 renders 11β-HSD1 as a primary oxidase [33].

The different directionality of 11β-HSD1 may play many physiological roles. It is abundantly present in human and rodent liver and acts as a predominant reductase, which generates active glucocorticoid locally [6, 34]. The glucocorticoid has been found to profoundly regulate liver the metabolism of glucose, which provides the crucial energy for all cells [35]. In the testis, 11β-HSD1 exclusively resides in Leydig cells [5], and its activity in Leydig cells is much higher than that of liver cells per se [7]. 11β-HSD1 is a bidirectional oxido-reductase [6]. However, in Leydig cells, 11β-HSD1 is a primary oxidase [6]. 11β-HSD1 has been suggested to protect this cell type from glucocorticoid-mediated inhibition of testosterone production by inactivating the active glucocorticoid [6, 18].

In conclusion, of the availability of H6PDH activity in liver and Leydig cells may explain the directionality of 11β-HSD1 in these cell types with a predominant reductase in liver cells and primary oxidase in Leydig cells.

## Supporting Information

S1 Fig Western blot for 11β-HSD1 from rat and human microsomes. A34 KD band corresponding to 11β-HSD1 was detected in all microsomes from rat liver (L) and Leydig cells (LC) as well as human liver (L) and testis (Tes). M = marker.
(TIF)

S2 Fig 11β-HSD1 reductase activities in rat liver and Leydig cell microsomes vs different concentrations of G6P. Rat liver microsome (2 μg) or Leydig cell microsome (0.3 μg) was incubated with 25 nM 11-dehydrocorticosterone for 30 min. The percentage of conversion of corticosterone into 11-dehydrocorticosterone was calculated. G6P dose-dependently increased the activity of 11β-HSD1 reductase in the liver microsome.
(TIF)

S3 Fig Effects of S3484 on 11β-HSD1 oxidase and reductase activities in rat liver microsomes. Rat liver microsome (2 μg) was permeabilized with 0.04 mg/ml alamethicin and incubated with 25 nM corticosterone or 11-dehydrocorticosterone in presence of 100 μM S3484 for 30 min. The percentage of conversion of corticosterone into 11-dehydrocorticosterone or reverse was calculated. S3484 did not affect the activity of 11β-HSD1 oxidase and reductase in the permeabilized liver microsome.
(PDF)

S1 Table NADPH and NADP+ concentrations in both Leydig and liver cells. (DOCX)
