# Supplementary figures and images for: Metabolic Coupling Determines the Activity: Comparison of 11β-Hydroxysteroid Dehydrogenase 1 and Its Coupling between Liver Parenchymal Cells and Testicular Leydig Cells
Source: PLoS One. 2015 Nov 3;10(11):e0141767. doi: 10.1371/journal.pone.0141767 (PMC4631333; doi:10.1371/journal.pone.0141767)

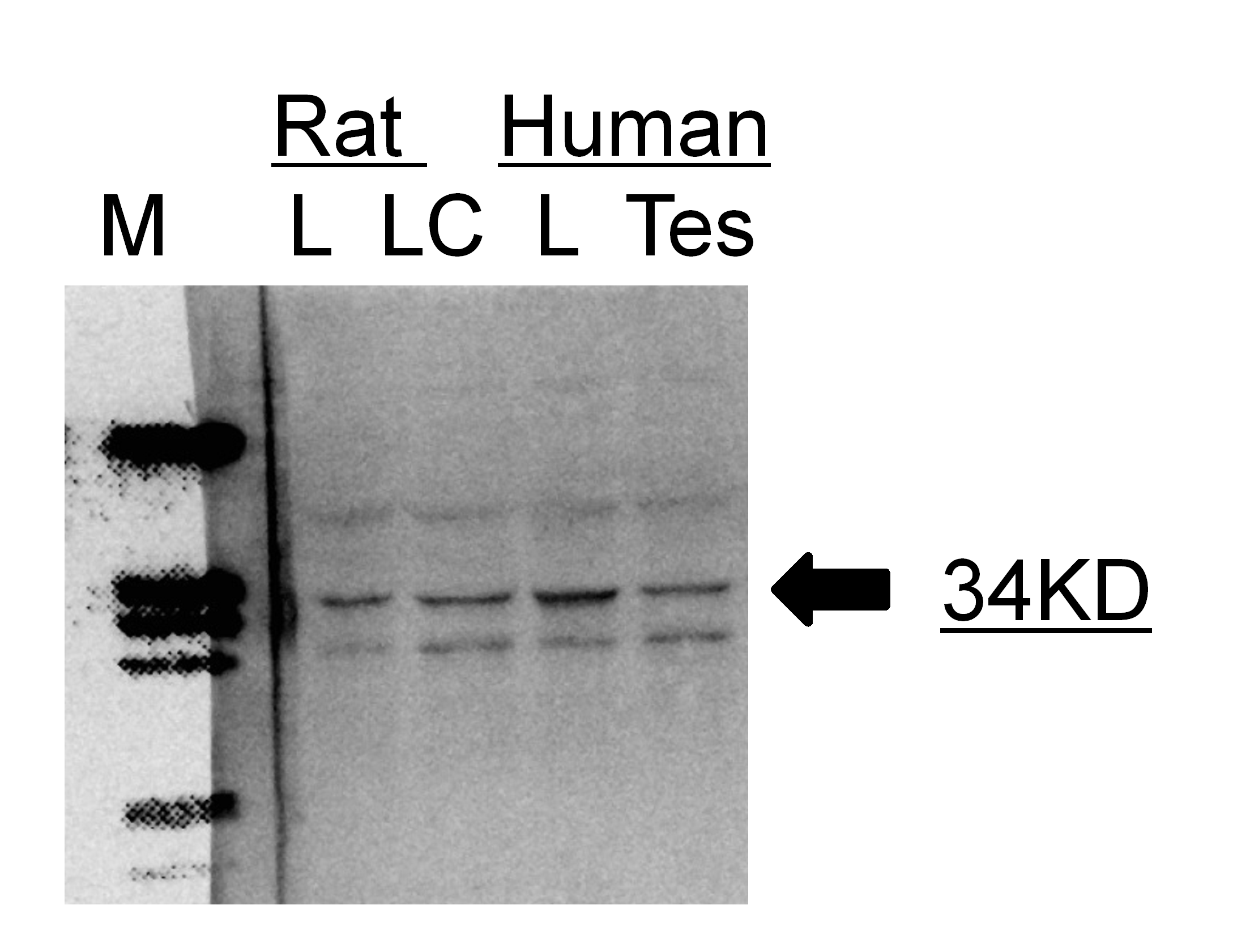

Supplement: S1 Fig — A34 KD band corresponding to 11β-HSD1 was detected in all microsomes from rat liver (L) and Leydig cells (LC) as well as human liver (L) and testis (Tes). M = marker. (TIF) [file pone.0141767.s001.tif]

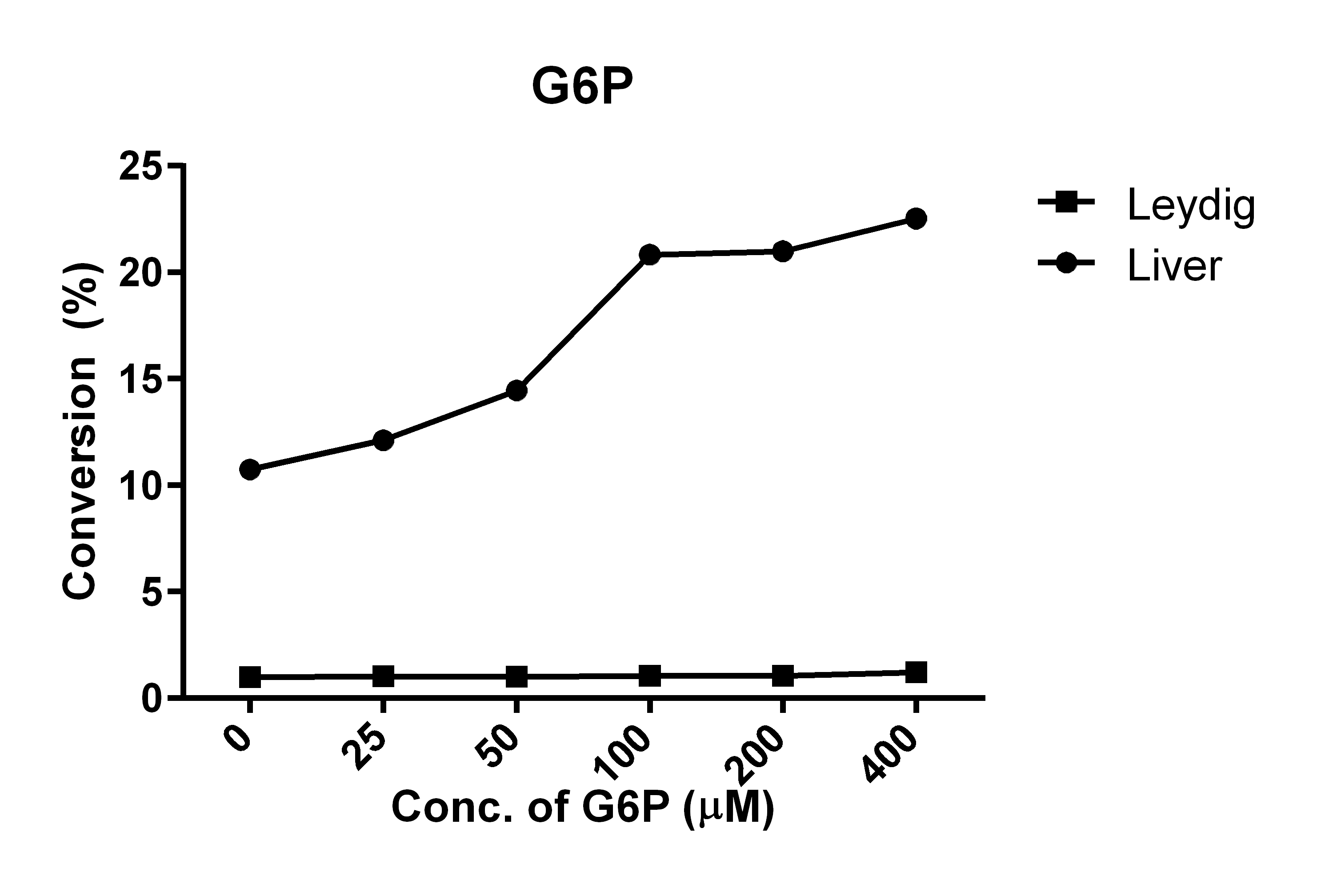

Supplement: S2 Fig — Rat liver microsome (2 μg) or Leydig cell microsome (0.3 μg) was incubated with 25 nM 11-dehydrocorticosterone for 30 min. The percentage of conversion of corticosterone into 11-dehydrocorticosterone was calculated. G6P dose-dependently increased the activity of 11β-HSD1 reductase in the liver microsome. (TIF) [file pone.0141767.s002.tif]

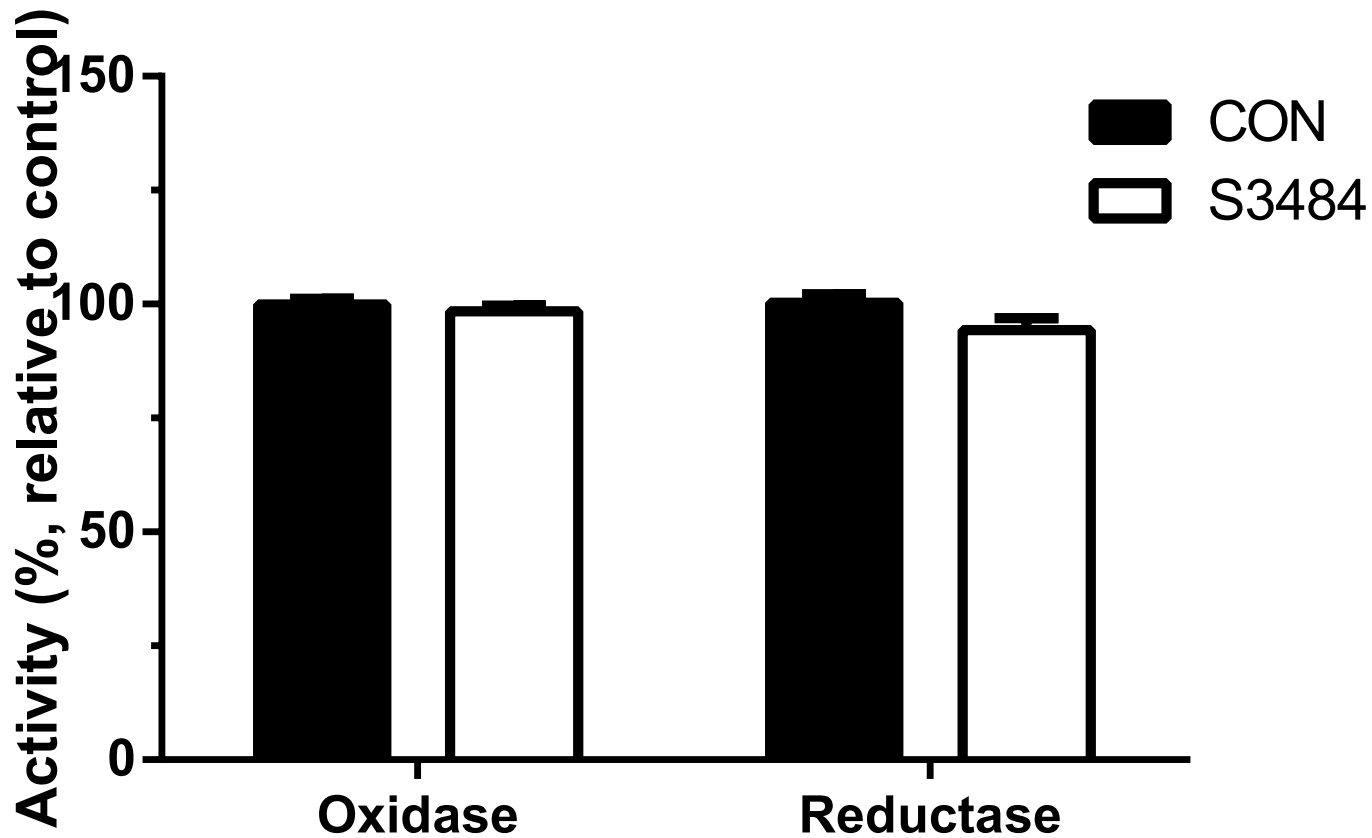

Supplement: S3 Fig — Rat liver microsome (2 μg) was permeabilized with 0.04 mg/ml alamethicin and incubated with 25 nM corticosterone or 11-dehydrocorticosterone in presence of 100 μM S3484 for 30 min. The percentage of conversion of corticosterone into 11-dehydrocorticosterone or reverse was calculated. S3484 did not affect the activity of 11β-HSD1 oxidase and reductase in the permeabilized liver microsome. (PDF) [file pone.0141767.s003.pdf]
