# Supplementary material for: Metabolic Coupling Determines the Activity: Comparison of 11β-Hydroxysteroid Dehydrogenase 1 and Its Coupling between Liver Parenchymal Cells and Testicular Leydig Cells
Source: PLoS One. 2015 Nov 3;10(11):e0141767. doi: 10.1371/journal.pone.0141767 (PMC4631333; doi:10.1371/journal.pone.0141767)
Supplement: S1 Table — (DOCX) [file pone.0141767.s004.docx]

**S1_Table . NADPH and NADP+ concentrations in both Leydig and liver cells.**

| **Cell type** | **NADPH**  **(nmol/10^6^ cells)** | **NADP+**  **(nmol/10^6^ cells)** | **NADP+/NADPH** |
| --- | --- | --- | --- |
| **Leydig cell** | 1.238 ± 0.036 | 0.926 ± 0.0978 | 0.673 ± 0.004 |
| **Liver cell** | 1.310 ± 0.037 | 0.992± 0.036 | 0.811 ± 0.0377 |

Mean ± SEM, n =3. There is no significant difference between Leydig and liver cells at each column.
